# Supplementary material for: Neuronal miR-9 promotes HSV-1 epigenetic silencing and latency by repressing Oct-1 and Onecut family genes
Source: Nat Commun. 2024 Mar 5;15:1991. doi: 10.1038/s41467-024-46057-6 (PMC10914762; doi:10.1038/s41467-024-46057-6)
Supplement: Supplementary file 1 — Supplementary Information File [file 41467_2024_46057_MOESM1_ESM.pdf]

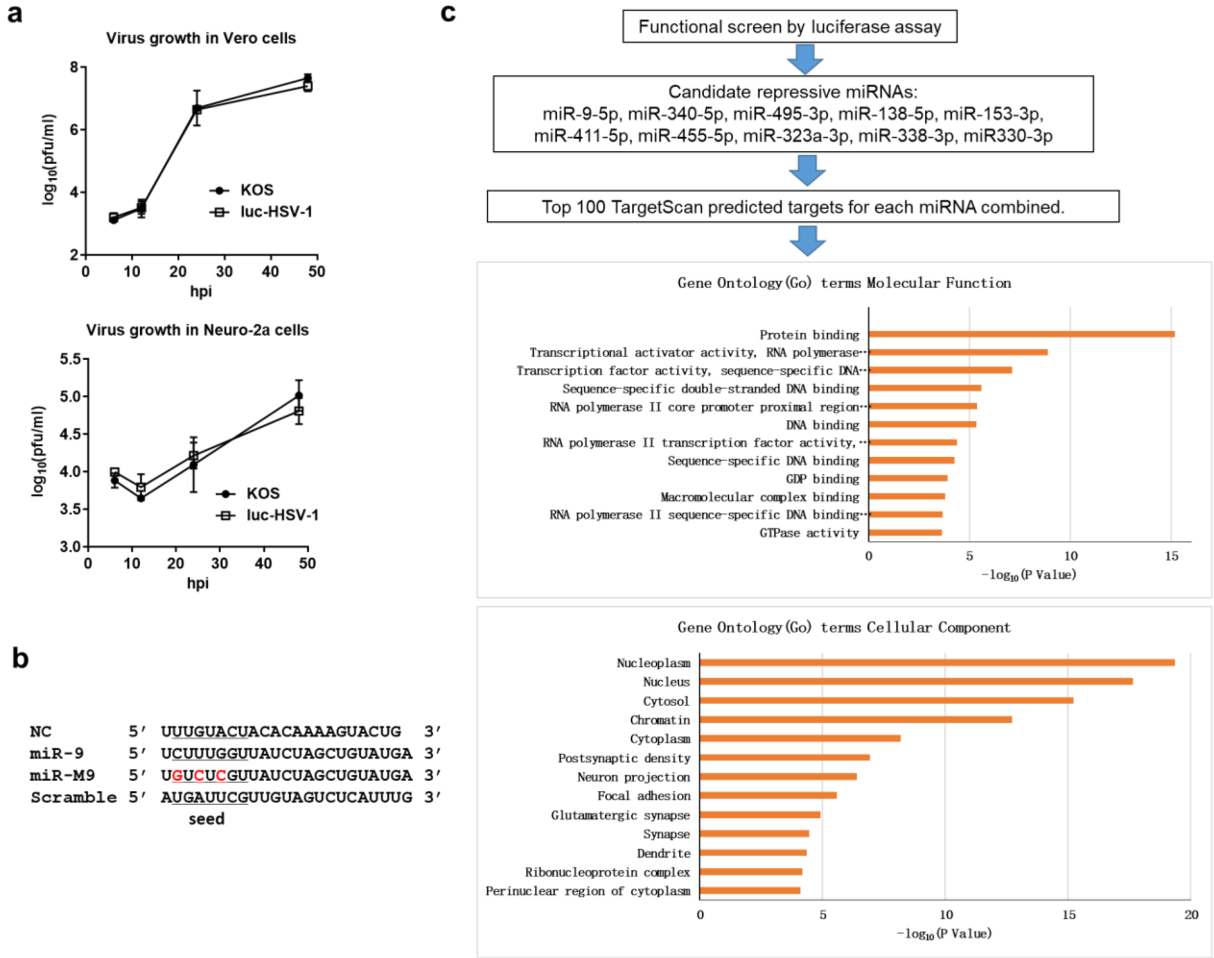

Supplementary Fig. 1. Additional data for screening of neuron-specific miRNAs regulating HSV-1 infection. a Growth curves of KOS and luc-HSV-1 in Vero (upper, MOI = 0.02) and Neuro-2a (lower, MOI = 0.1) cells. *n* = 3 biologically independent samples. Data are presented as the mean ± s.d. **b** Sequences of miR-9 and control mimics used for transfection experiments. The seed regions are underlined. **c** Gene ontology (GO) analysis of enriched molecular functions and cellular components of the putative targets of the candidate miRNAs predicted by TargetScan. The procedure for candidate selection is outlined above the GO results. Source data are provided as a Source Data file.

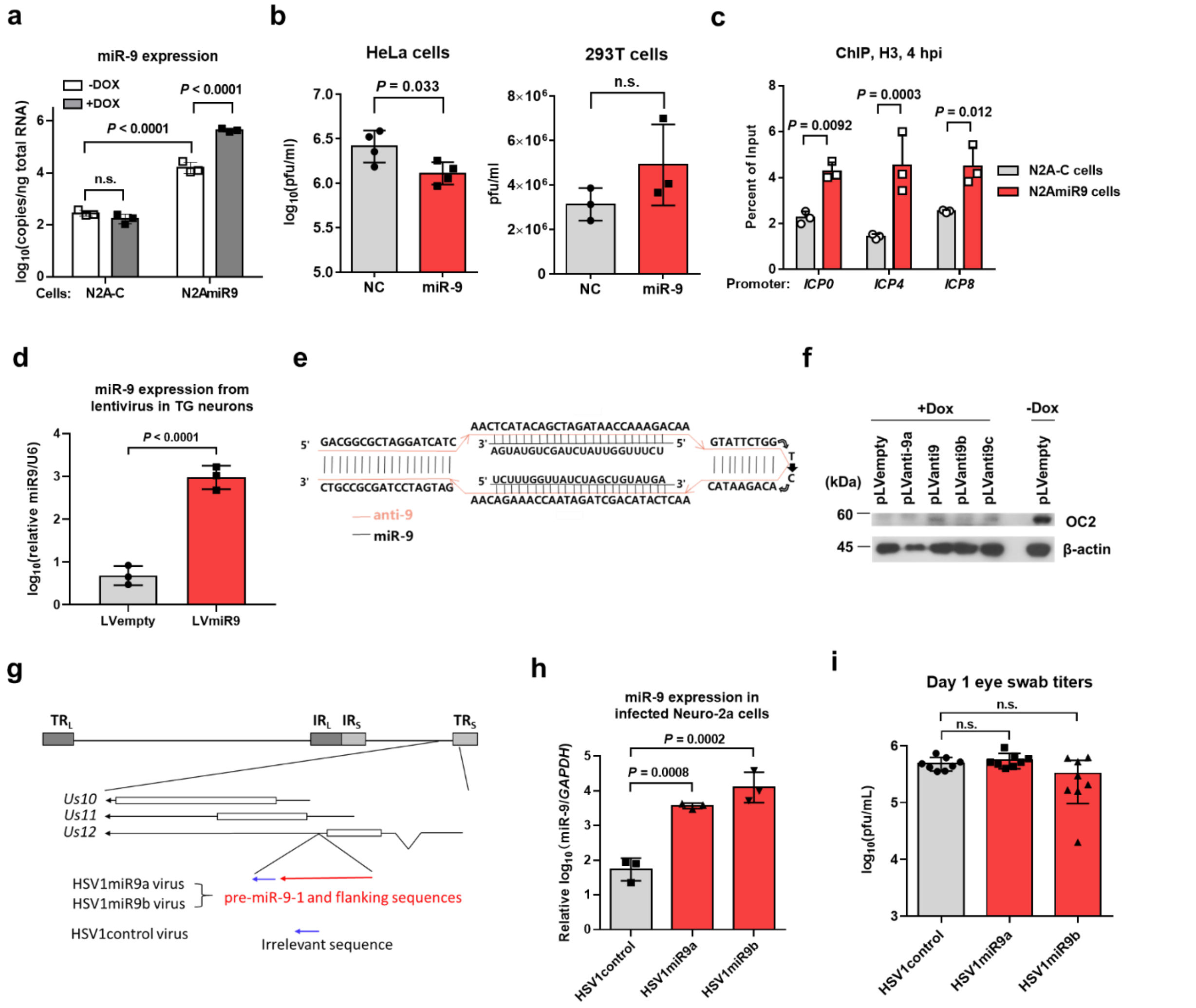

Supplementary Fig. 2. Additional data for miR-9 functions during HSV-1 infection. a miR-9 levels in the indicated cell lines are quantified by qRT-PCR. **b** HeLa (left) or 293T (right) cells were transfected with 20 nM negative control (NC) or miR-9 mimic for 24 h and then infected with KOS (MOI of 0.3 for HeLa cells and 0.01 for 293T cells) for 48 h before virus titration. **c** N2A-C or N2AmiR9 cells maintained in 1 μg/ml Dox were infected with HSV-1 (MOI = 1) for 4 h before ChIP-qPCR analysis of the enrichment of histone H3 on the indicated promoters. **d** Mouse TG neurons were transduced with the indicated lentivirus and then miR-9 levels were quantified by qRT-PCR. **e** miR-9 and anti9 sequences and the base pairs between them. **f** N2AmiR9 cells were treated or not treated with 1 μg/ml Dox for 48 h, then transfected with the empty plasmid or a plasmid expressing one of four different miR-9 antisense sequences before analysis of OC2 expression by Western blots. LVanti9 was used for other experiments. **g** Schematic of recombinant viruses showing insertion of miR-9 expressing or control sequences between US11 and US12 open reading frames. **h** Neuro-2a cells were infected with the viruses indicated at the bottom (MOI = 10) for 8 h before being harvested for quantification of miR-9 by qRT-PCR. **i** Mice were inoculated on the cornea with a dose of 2 x 10^5 pfu/eye and viral titers in tear film at 1 dpi were quantified by plaque assays. *n* = 3 (**a, c, d, h**, right of **b**), 4 (left of **b**) or 8 (**i**) biologically independent samples. Data were analyzed by two-sided unpaired t tests (**b, d**), two-way ANOVA with Sidak's multiple comparisons tests (**a, c**) or one-way ANOVA with Dunnett's multiple comparisons tests (**h, i**) and are presented as the mean ± s.d. Source data are provided as a Source Data file.

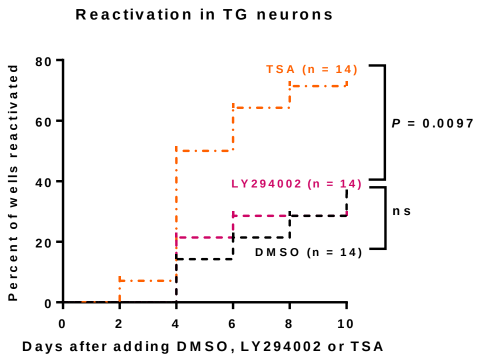

Supplementary Fig. 3. Validation of primary neuronal model of HSV-1 latency and reactivation. In the latency-reactivation model in mouse TG neurons (Fig. 2d), after the addition of 1:1000 volume of DMSO, TSA (0.2 μM) or LY294002 (20 μM), supernatants were collected every two days for assays of infectious virus positivity. The sample sizes are indicated on the figure. Data were analyzed by Log-rank (Mantel-Cox) tests. Source data are provided as a Source Data file.

a

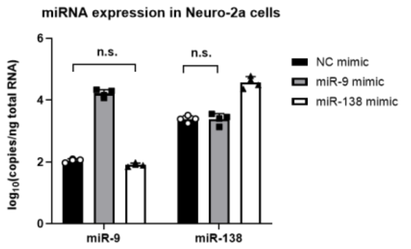

b

```
3'          AGUAUGUCGAUCUAUUGGUUUCU 5' miR-9
5' AAUCGUGUUGAUGACACCAAAGAUACCAAAGA 3' Human OC2 sites 7+8
3' AGUAUGUCGAUCUAUUGGUUUCU 5' miR-9
```

c

mRNA	293T cells		Neuro-2a cells	
	Human sites	Reads	Mouse sites	Reads
OC2	Site 1	4	Site 1	9
	Site 2	0	Site 2	0
	Site 3	173	Site 3	3
	Site 4	206	Site 4	10
	Site 5	0	Site 5	0
	Site 6	6	Site 6	0
	Site 7+8	1703	Site 7+8	34
Oct-1	Site 1	0	Site 1	0
	Site 2	50	Site 2	13
	Site 3	0	Site 3	0
	Site 4	165	Site 4	1
			Site 5	50

d

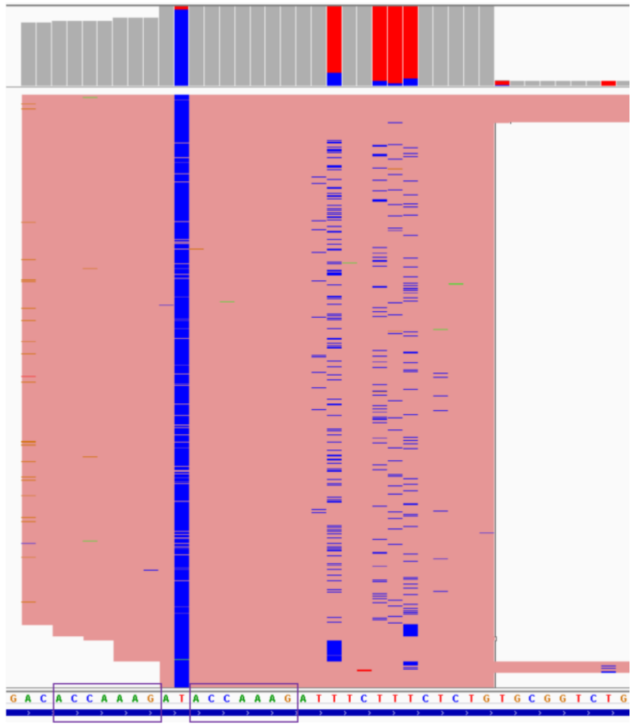

Supplementary Fig. 4. Additional data about miR-9 targets. a Neuro-2a cells were transfected with 20 nM of the indicated miRNA mimic for 48 h before quantification of miR-9 and miR-138 levels. Data were analyzed by two-way ANOVA with Sidak's multiple comparisons tests and detected no significant difference between the compared groups indicated by brackets. *n* = 4 biologically independent samples. **b** Base pairing between miR-9 and human OC2 sites 7+8. **c** PAR-CLIP reads aligned to OC2 or Oct-1 3' UTRs in 293T and Neuro-2a cells according to data obtained from our previous publication[28]. **d** Reads aligned to human OC2 Sites 7+8 in 293T cells. At the top is the coverage plot in which when the frequency of a variant is greater than 3%, the identity of the nucleotide is displayed in color with red representing T and blue representing C. In the middle, stacked reads are displayed with T to C mutations shown in blue. At the bottom are sequences with the positions of the two miR-9 seed binding sites (see panel **b**) highlighted in purple boxes. Source data are provided as a Source Data file.

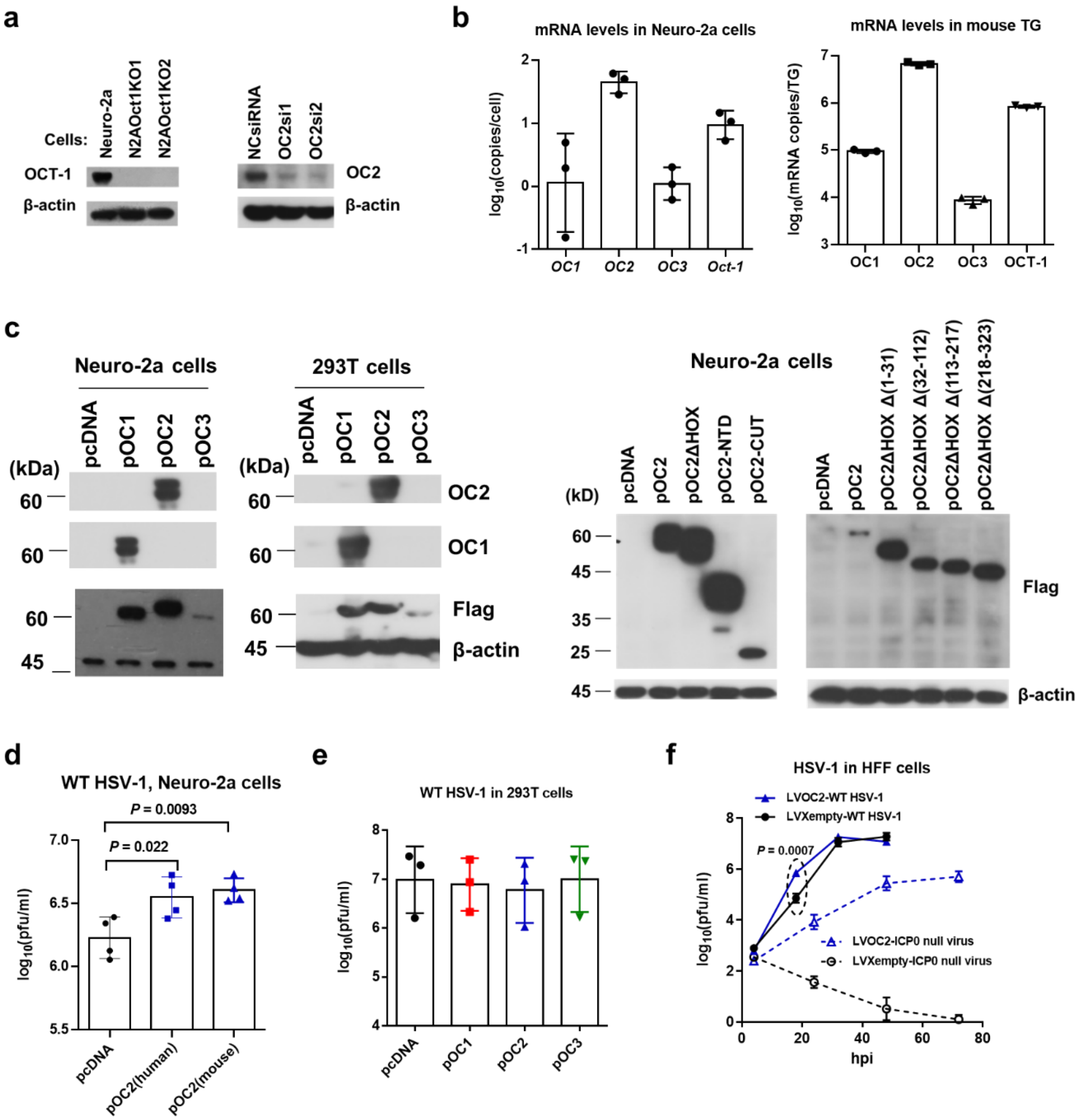

Supplementary Fig. 5. Additional data for effects of OC proteins on HSV-1 replication. a Western blot analysis of OCT-1 expression from the indicated cells (left) or OC2 expression after Neuro-2a cells were transfected with 80 nM siRNA for 48 h. **b** mRNA levels of the indicated genes in Neuro-2a cells (left) or mouse TG (right) were estimated by qRT-PCR using DNA standards assuming reverse transcription was efficient. **c** Neuro-2a cells were transfected with 200 ng/ml of the indicated plasmids expressing Flag-tagged OC proteins for 48 h before western blot analysis using the antibodies indicated to the right of the blots. **d** Neuro-2a cells were transfected with 200 ng/ml plasmid, then infected with HSV-1 (MOI = 0.2) for 48 h before virus titration. **e** 293T cells were transfected with 200 ng/ml plasmid for 48 h, then infected with HSV-1 (MOI = 0.1) for 48 h before virus titration. **f** HFF cells were transduced with the indicated lentiviruses for 72 h, then infected with KOS or 7134 (ICP0-null) virus (MOI = 0.05) for the indicated times before virus titration. *n* = 3 (**b, e, f**) or 4 (**d**) biologically independent samples. Data were analyzed by one-way ANOVA with Dunnett's multiple comparisons tests (**d, e**) or two-sided unpaired t tests (**f**) and are presented as the mean ± s.d. Source data are provided as a Source Data file.

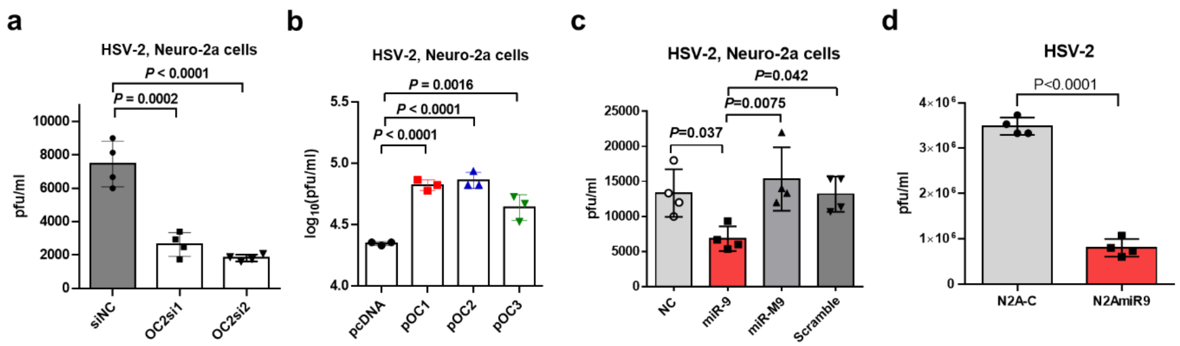

Supplementary Fig. 6. Regulation of HSV-2 replication by miR-9 and OC proteins. **a** Neuro-2a cells were transfected with 80 nM siRNA for 40 h and then infected with HSV-2 strain 186 (MOI = 0.2) for 48 h before virus titration. **b** Neuro-2a cells were transfected with 200 ng/ml plasmid for 24 h and then infected with HSV-2 (MOI = 0.2) for 48 h before virus titration. **c** Neuro-2a cells were transfected with 40 nM miRNA mimic for 24 h and then infected with HSV-2 (MOI = 0.2) for 48 h before virus titration. **d** The indicated cell lines were infected with HSV-2 (MOI = 1) for 48 h before virus titration. *n* = 3 (**b**) or 4 (**a, c, d**) biologically independent samples. Data were analyzed by one-way ANOVA with Dunnett's multiple comparisons tests and are presented as the mean ± s.d. Source data are provided as a Source Data file.

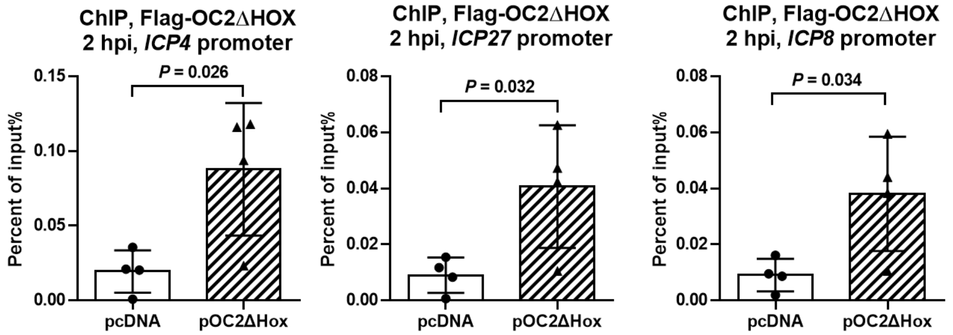

Supplementary Fig. 7. OC2ΔHOX binds to HSV-1 lytic gene promoters at 2 hpi. Neuro-2a cells were transfected with 400 ng/ul of the indicated plasmids for 40 h and then infected with ICP0-null virus (MOI = 3) for 2 h before ChIP-qPCR analysis of the enrichment of Flag-tagged OC2ΔHOX at the indicated promoters using a Flag antibody. *n* = 4 biologically independent samples. Data were analyzed by two-sided unpaired t tests and are presented as the mean ± s.d. Source data are provided as a Source Data file.

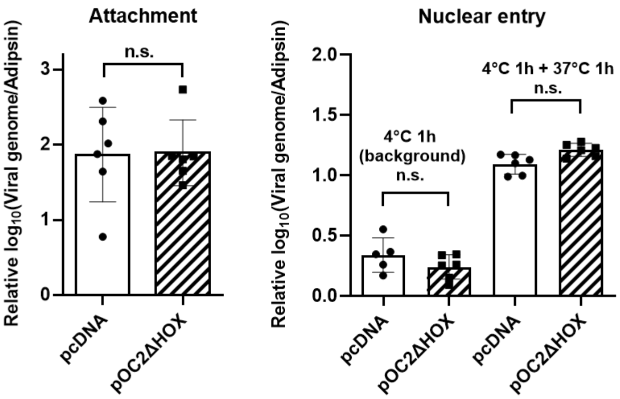

Supplementary Fig. 8. No effect of OC2ΔHOX on virus attachment and nuclear entry. Neuro-2a cells were transfected with 200 ng/ml plasmid for 42 h and then incubated with the ICP0-null virus (MOI = 1) at 4°C for 1 h. To analyze attachment (left), the cells were washed by PBS before qPCR analysis of viral genomes. To analyze nuclear entry (right), cells were either immediately harvested (for background analysis) or harvested after additional incubation at 37°C for 1 h before isolation of nuclear fractions and qPCR analysis of viral genomes in the nucleus. *n* = 6 biologically independent samples. Data were analyzed by two-sided unpaired t tests and are presented as the mean ± s.d. n.s., non-significant. Source data are provided as a Source Data file.

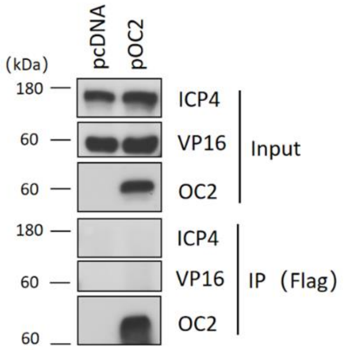

Supplementary Fig. 9. No evidence that OC2 interacts with VP16 or ICP4. Neuro-2a cells in each 100-mm plate were transfected with 4 µg of an empty vector (pcDNA) or a plasmid expressing Flag-tagged OC2 (pOC2) for 36 h and then infected with KOS (MOI = 5) for 12 h. After the cells were lysed, the indicated proteins were immunoprecipitated by an anti-Flag antibody. The lysates (input) and immunoprecipitated samples (IP) were analyzed by Western blots using OC2, ICP4 and VP16 antibodies. Source data are provided as a Source Data file.

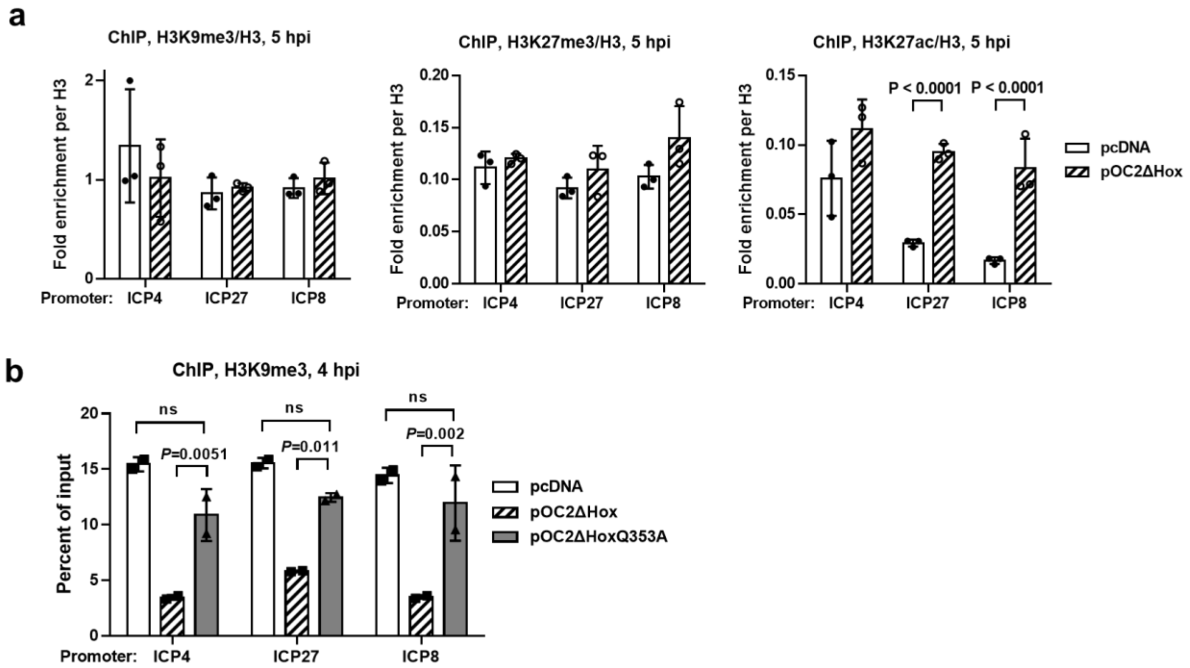

Supplementary Fig. 10. Additional ChIP-qPCR data for the effects of OC2ΔHOX on viral chromatin. a Re-analysis of the 5-h data in the Fig. 7a experiment. Enrichment of histone H3 modifications were normalized to H3. **b** Neuro-2a cells were transfected with 600 ng/ml plasmid for 42 h and then infected with ICP0-null virus (MOI = 2) for 4 h before ChIP-qPCR analysis of the enrichment of H3K9me3 on the indicated viral gene promoters. *n* = 2 (**b**) or 3 (**a**) biologically independent samples. Data were analyzed by two-way ANOVA with Sidak's multiple comparisons tests and are presented as the mean ± s.d. Source data are provided as a Source Data file.

Supplementary Table 1. Synthetic DNA sequences.

kana-ICP47-miR-9	AATCCGGTAACCCGTTGAGTCCCGGGTACGACCATCACCCGAGTCTCTGGCTTCTGAGGCGGAAAGAACCAG<br>CTGGGGCTCTAGGGGGTAGCGGCCGCTAGGGATAACAGGGTAATCGATTTATTCAACAAAGCCACGTTGTG<br>TCTCAAAATCTCTGATGTTACATTGCACAAGATAAAAATATATCATCATGAACAATAAAACTGTCTGCTTACAT<br>AAACAGTAATACAAGGGGTGTTATGAGCCATATTCAACGGGAAACGTCTTGCTCGAGGCCGCGATTAAATTC<br>CAACATGGATGCTGATTTATATGGGTATAAATGGGCTCGCGATAATGTCGGGCAATCAGGTGCGACAATCTA<br>TCGATTGTATGGGAAGCCCGATGCGCCAGAGTTGTTTCTGAAACATGGCAAAGGTAGCGTTGCCAATGATGT<br>TACAGATGAGATGGTCAGACTAAACTGGCTGACGGAATTTATGCCTCTTCCGACCATCAAGCATTTTATCCGT<br>ACTCCTGATGATGCATGGTTACTCACCACTGCGATCCCCGGGAAAACAGCATTCCAGGTATTAGAAGAATAT<br>CCTGATTCAGGTGAAAATATTGTTGATGCGCTGGCAGTGTTCCTGCGCCGGTTGCATTCGATTCCTGTTTGTA<br>ATTGTCCTTTTAACAGCGATCGCGTATTTCGTCTCGCTCAGGCGCAATCACGAATGAATAACGGTTTGGTTGA<br>TGCGAGTGATTTTGATGACGAGCGTAATGGCTGGCCTGTTGAACAAGTCTGGAAAGAAATGCATAAGCTTTT<br>GCCATTCTCACCGGATTCAGTCGTCACTCATGGTGATTTCTCACTTGATAACCTTATTTTTGACGAGGGGAAA<br>TTAATAGGTTGTATTGATGTTGGACGAGTCGGAATCGCAGACCGATACCAGGATCTTGCCATCCTATGGAAC<br>TGCCTCGGTGAGTTTTCTCCTTCATTACAGAAACGGCTTTTTCAAAAATATGGTATTGATAATCCTGATATGAA<br>TAAATTGCAGTTTCATTTGATGCTCGATGAGTTTTTCTAATCAGAATTGGTTAATTGGTTGTAACACTGGCCTT<br>CTGAGGCGGAAAGAACCAGCTGGGGCTCTAGGGGGTAGAATTCCCTGGACGACCACTCTTCGGTACTGCCA<br>GAAAGGATCAGGACCTGGAGTCTGGCAAGAGGAAGACAGAGGCCTGTGTGGGAAGCGAGTTGTTATCTTT<br>GGTTATCTAGCTGTATGAGTGTATTGGTCTTCATAAAGCTAGATAACCGAAAGTAAAAACTCCTTCAAGATCG<br>CCGGGGAGCGTGTGAGAATGAAAGACTACAGCCGAAAGCTTAAGGCGGAGGGTGGTTCCCCCCCGTGTCTC<br>TCGAGATGAGCCAGACCCAACCC
kana-ICP47-Control	AATCCGGTAACCCGTTGAGTCCCGGGTACGACCATCACCCGAGTCTCTGGCTTCTGAGGCGGAAAGAACCAG<br>CTGGGGCTCTAGGGGGTAGCGGCCGCTAGGGATAACAGGGTAATCGATTTATTCAACAAAGCCACGTTGTG<br>TCTCAAAATCTCTGATGTTACATTGCACAAGATAAAAATATATCATCATGAACAATAAAACTGTCTGCTTACAT<br>AAACAGTAATACAAGGGGTGTTATGAGCCATATTCAACGGGAAACGTCTTGCTCGAGGCCGCGATTAAATTC<br>CAACATGGATGCTGATTTATATGGGTATAAATGGGCTCGCGATAATGTCGGGCAATCAGGTGCGACAATCTA<br>TCGATTGTATGGGAAGCCCGATGCGCCAGAGTTGTTTCTGAAACATGGCAAAGGTAGCGTTGCCAATGATGT<br>TACAGATGAGATGGTCAGACTAAACTGGCTGACGGAATTTATGCCTCTTCCGACCATCAAGCATTTTATCCGT<br>ACTCCTGATGATGCATGGTTACTCACCACTGCGATCCCCGGGAAAACAGCATTCCAGGTATTAGAAGAATAT<br>CCTGATTCAGGTGAAAATATTGTTGATGCGCTGGCAGTGTTCCTGCGCCGGTTGCATTCGATTCCTGTTTGTA<br>ATTGTCCTTTTAACAGCGATCGCGTATTTCGTCTCGCTCAGGCGCAATCACGAATGAATAACGGTTTGGTTGA<br>TGCGAGTGATTTTGATGACGAGCGTAATGGCTGGCCTGTTGAACAAGTCTGGAAAGAAATGCATAAGCTTTT<br>GCCATTCTCACCGGATTCAGTCGTCACTCATGGTGATTTCTCACTTGATAACCTTATTTTTGACGAGGGGAAA<br>TTAATAGGTTGTATTGATGTTGGACGAGTCGGAATCGCAGACCGATACCAGGATCTTGCCATCCTATGGAAC<br>TGCCTCGGTGAGTTTTCTCCTTCATTACAGAAACGGCTTTTTCAAAAATATGGTATTGATAATCCTGATATGAA<br>TAAATTGCAGTTTCATTTGATGCTCGATGAGTTTTTCTAATCAGAATTGGTTAATTGGTTGTAACACTGGCCTT<br>CTGAGGCGGAAAGAACCAGCTGGGGCTCTAGGGGGTAGAATTCAAGCTTAAGGCGGAGGGTGGTTCCCCC<br>CCGTGTCTCTCGAGATGAGCCAGACCCAACCC

Supplementary Table 2. Sequences of primers used to construct plasmids and recombinant viruses.

Primer names	Primer sequences
AgeLucKan Fw	ATTTTAACCGGTAAGACACTGGGTGTGAACCTAGGGATAACAGGGTAATCGATT
AgeLucKan Rv	ACCACAACCGGTGTGTCCACCACCTTAGCCAGTGTTACAACCAATTAACC
gCLuc2-FOR	TCTCGCTTTGCCGGGAACGCTAGCCGATCCCTCGCGAGGGGGAGGCGTCGGGCATGGAAGATGCC<br>AAAAACATTAAG
gCLuc2-REV	GGAAGAGAGGGTGGCGGCTTTATAGCGCCCAGCGGTGGGCGGGATAGAGGTACCACATTTGTAG<br>AGGTTTTAC
pTRIPZ-miR9-F	CCGCTCGAGCCTGGACGACCACTCTTCG
pTRIPZ-miR9-R	CGACGCGTTCGGCTGTAGTCTTTCATTCTCAC
miR-TransformF	CGTTGAGTCCCGGGTAC
miR-TransformR	GGGTCTGGCTCATCTCG
OC1-3UTR-F	CCGCTCGAGAGGAAGAACCACAAACTAAAACCT
OC1-3UTR-R	AAATATGCGGCCGCTTTTGGGGGTAATATATACTAGCAACAAAT

OC2-3UTR-F	CCGCTCGAGCTCTTCTGTTGTTGAATCATCCTTGC
OC2-3UTR-R	AAATATGCGGCCGCAGCAATCACATGAAAACGAGGCT
OC3-3UTR-F	CCGCTCGAGACCTCACTCAAGCTCATGTCT
OC3-3UTR-R	AAATATGCGGCCGCGCTGGAGGTGTTGATCCCT
Oct1-3UTR-F	CCGCTCGAGTGATCCCACAGAACTTAAGTGGGA
Oct1-3UTR-R	AAATATGCGGCCGCCTGAGGTTAGACTAATGCAAGTGCT
lvx-OC1-F	CTAGTCTAGAATGAACGCGCAGCTGACCAT
lvx-OC1-R	TCGCGGATCCTCATGCTTTGGTACAAGTGCT
lvx-OC2-F	CTAGTCTAGAATGAAGGCTGCCTACACCG
lvx-OC2-R	TCGCGGATCCTCATGCTTTGGTACACGTGCTGG
lvx-OC3-F	CTAGTCTAGAATGGAGCTGAGCCTGGAGAGC
lvx-OC3-R	TCGCGGATCCTCAGGCCTTGGAGAAAGTGG
OC2ΔHOXΔCUT-F	CAAGACGATCTGAGCACAG
OC2ΔHOXΔCUT-R	CTGCGAGCCCGATGAGGA
OC2ΔHOX-F	CAAGACGATCTGAGCACAG
OC2ΔHOX-R	GGAATTGTTCCTGTCTTTG
Δ(94-336)-F	ATCCTGGACGGCGGCGAC
Δ(94-336)-R	AGTGCCCAGACTTTCCATTGTCAG
Δ(337-651)-F	CCCGGCATGAGCCAGAGC
Δ(337-651)-R	CGAGGCCATGCTGGTGAC
Δ(652-969)-F	GTGGCCACGTCGGGCCAGCTGGAAGAAAT
Δ(652-969)-R	CATCTCCTTGTAGGGACTGTAGAGGTTG
Δ(1-93)-F	CCGGAATTCATGTTGCACGGGCCGGCCGG
Δ(1-93)-R	TCGCGGATCCTCATGCTTTGGTACACGTGCTGG
CUT-F	GTGGCCACGTCGGGCCAGCTGGAAGAAAT
CUT-R	CATGAATTCTGCAGATATCCAGCACAGTGGCGG
NTD-F	GGATCCGAGCTCGGTACCAAGCTTAA
NTD-R	CTGCGAGCCCGATGAGGA
Q353A-F	CAGTATCCCCGCGGCGATCTTTGCGC
Q353A- R	TAGCGCTTCAGCTCCGCT
S364A-F	GCTGTGCCGGGCGCAGGGGACTC
S364A-R	ACCCTCTGCGCAAAGATCGC
Q365A-F	GTGCCGGTCTGCGGGGACTCTCTC
Q365A-R	AGCACCCTCTGCGCAAAG
T367A-F	GTCTCAGGGGGCACTCTCCGACC
T367A-R	CGGCACAGCACCCTCTGC
S369A-F	GGGGACTCTCGCCGACCTGCTCC
S369A-R	TGAGACCGGCACAGCACC

Supplementary Table 3. siRNA sequences.

siRNA names	siRNA target sequences
NCsiRNA	UUCUCCGAACGUGUCACGU
OC2si1	CAUGGGCAUGAGCAACACCUA
OC2si2	CCGAACACUCUUCGCCAUCUU

Supplementary Table 4. qPCR primer sequences.

Primer names	Primer sequences
ICP0mRNA-F	AGCGAGTACCCGCCGGCCTG
ICP0mRNA-R	CAGGTCTCGGTCGCAGGGAAAC
ICP27mRNA-F	GTGTGCAGCCGTGTTCCAA
ICP27mRNA-R	AGCGACCGGGCCCGAATC
TKmRNA-F	ACCCGCTTAACAGCGTCAACA
TKmRNA-R	CCAAAGAGGTGCGGGAGTTT
gCmRNA-F	GCCCATTTCGTACGACTACA
gCmRNA-R	GGTGCTCTAGAACGGGAATC
qmGAPDH-F	GAAGGTCGGTGTGAACGGATT
qmGAPDH-R	GCCTTGACTGTGCCGTTGAA
ChIP-ICP0-F	CGCCTTCCCGAAGAAACTCA
ChIP-ICP0-R	CGCTCAATGAACCCGCATT
ChIP-ICP4-F	CGCATGGCATCTCATTACCG
ChIP-ICP4-R	TAGCATGCGGAACGGAAGC
ChIP-ICP8-F	GAGACCGGGGTTGGGGAATGAATC
ChIP-ICP8-R	CCCCGGGGGTTGTCTGTGAAGG
ChIP-ICP27-F	ACCCAGCCAGCGTATCCACC
ChIP-ICP27-R	ACACCATAAGTACGTGGCATGT
ChIP-GAPDH-F	CAGGCGCCCAATACGACCAAAATC
ChIP-GAPDH-R	TTCGACAGTCAGTCAGCCGCATCTTCTT
qmOC1-F	CCCAAGCCCTGGAGCAAACT
qmOC1-R	GCCTCTGTCCTTCCCGTGTT
qmOC2-F	AACGCAAAGAGCAAGAACCAA
qmOC2-R	AAGATGGCGAAGAGTGTTCGG
qmOC3-F	CCCTGGAGCAAGCTCAAATCC
qmOC3-R	GCTGATCCTGCTCTTTACGCT
qmOCT1-F	CGGAGCCAGCCAGGTGAT
qmOCT1-R	AGCAGCCATAGCAGCAAGACT
qhOC1-F	CCCAAGCCCTGGAGCAAACT
qhOC1-R	GCCTCTGTCCTTCCCGTGTT
qhOC2-F	AACGCAAAGAGCAAGAACCAA
qhOC2-R	AAGATGGCGAAGAGTGTTCGG
qhOC3-F	AAATCCGGCCGCGAGAC
qhOC3-R	GCTCCTGTTCCTTGCGCTT

qhGAPDH-F	GAAGGTCGGAGTCAACGGATT
qhGAPDH-R	GCCTTGACGGTGCCATGGAA
adipsin-F	TCCGGCAGCCCTCTAGT
adipsin-R	TAGGATGACACTCGGGTATAGAC
qLV-mcherry-F	GGCACCAACTTCCCCTCC
qLV-mcherry-R	TCTGCTTGATCTCGCCCTTC
