# Supplementary material for: Neuronal miR-9 promotes HSV-1 epigenetic silencing and latency by repressing Oct-1 and Onecut family genes
Source: Nat Commun. 2024 Mar 5;15:1991. doi: 10.1038/s41467-024-46057-6 (PMC10914762; doi:10.1038/s41467-024-46057-6)
Supplement: Supplementary file 2 — Reporting Summary [file 41467_2024_46057_MOESM2_ESM.pdf]

nature portfolio

Corresponding author(s): Dongli Pan

Last updated by author(s): Jan 22, 2024

Reporting Summary

Nature Portfolio wishes to improve the reproducibility of the work that we publish. This form provides structure for consistency and transparency in reporting. For further information on Nature Portfolio policies, see our Editorial Policies and the Editorial Policy Checklist.

Statistics

For all statistical analyses, confirm that the following items are present in the figure legend, table legend, main text, or Methods section.

n/a	Confirmed	
[ ]	[x]	The exact sample size (*n*) for each experimental group/condition, given as a discrete number and unit of measurement
[ ]	[x]	A statement on whether measurements were taken from distinct samples or whether the same sample was measured repeatedly
[ ]	[x]	The statistical test(s) used AND whether they are one- or two-sided<br>*Only common tests should be described solely by name; describe more complex techniques in the Methods section.*
[x]	[ ]	A description of all covariates tested
[ ]	[x]	A description of any assumptions or corrections, such as tests of normality and adjustment for multiple comparisons
[ ]	[x]	A full description of the statistical parameters including central tendency (e.g. means) or other basic estimates (e.g. regression coefficient) AND variation (e.g. standard deviation) or associated estimates of uncertainty (e.g. confidence intervals)
[ ]	[x]	For null hypothesis testing, the test statistic (e.g. *F*, *t*, *r*) with confidence intervals, effect sizes, degrees of freedom and *P* value noted<br>*Give P values as exact values whenever suitable.*
[x]	[ ]	For Bayesian analysis, information on the choice of priors and Markov chain Monte Carlo settings
[x]	[ ]	For hierarchical and complex designs, identification of the appropriate level for tests and full reporting of outcomes
[x]	[ ]	Estimates of effect sizes (e.g. Cohen's *d*, Pearson's *r*), indicating how they were calculated

Our web collection on statistics for biologists contains articles on many of the points above.

Software and code

Policy information about availability of computer code

Data collection	TargetScanHuman8.0 (https://www.targetscan.org/vert_80)<br>TissueAtlas (https://ccb-web.cs.uni-saarland.de/tissueatlas/)
Data analysis	GraphPad Prism version 8.0.2 for Windows (www.graphpad.com)<br>ImageJ version 1.52n (https://imagej.nih.gov/ij/)<br>QuantStudio Design&Analysis Software v1.5.1 (https://www.thermofisher.cn/cn/zh/home/global/forms/life-science/quantstudio-3-5-software.html)<br>PyMOL software (PDB code 2D5V) (https://pymol.org)<br>fastp (versions 0.20.0 and 0.23.1)(https://github.com/OpenGene/fastp)<br>BWA-MEM (https://github.com/lh3/bwa)<br>sambamba (https://github.com/biod/sambamba)<br>SAMtools (https://github.com/samtools/samtools)<br>Bowtie2 (versions 2.3.4.3, 2.2.5)(https://bowtie-bio.sourceforge.net/bowtie2/index.shtml)<br>DeepTools (version 3.5.1) (https://github.com/deeptools/deepTools/releases)<br>MACS2 (version 2.2.7.1) (https://pypi.org/project/MACS2)<br>ChIPseeker (version1.32.1) (https://bioconductor.org/packages/release/bioc/html/ChIPseeker.html)<br>Homer2 (version4.11.1) (https://homer-fnirs.org)<br>Integrative Genomics Viewer (verison2.16.2) (https://software.broadinstitute.org/software/igv/)<br>SOAPnuke (version1.5.2) (https://github.com/BGI-flexlab/SOAPnuke)

HISAT (version2.1.0) (http://ccb.jhu.edu/software/hisat/index.shtml)
RSEM (version1.3.1) (http://deweylab.github.io/RSEM/)
DESeq2 (version1.4.5) (https://www.bioconductor.org/packages/devel/bioc/vignettes/DESeq2/inst/doc/DESeq2.html)
DAVID Bioinformatics Resources(http://david.ncifcrf.gov/)

For manuscripts utilizing custom algorithms or software that are central to the research but not yet described in published literature, software must be made available to editors and reviewers. We strongly encourage code deposition in a community repository (e.g. GitHub). See the Nature Portfolio guidelines for submitting code & software for further information.

Data

Policy information about availability of data

All manuscripts must include a data availability statement. This statement should provide the following information, where applicable:

- Accession codes, unique identifiers, or web links for publicly available datasets
- A description of any restrictions on data availability
- For clinical datasets or third party data, please ensure that the statement adheres to our policy

The ChIP-seq, RNA-seq and ATAC-seq data have been deposited to the Gene Expression Omnibus under accession numbers GSE241863, GSE241029 and GSE240221, respectively. The PAR-CLIP data from our previous study are available at Gene Expression Omnibus under accession number GSE127503. The HSV-1 KOS strain genome sequence and annotation were obtained from GenBank under accession number JQ673480.1. Mouse (mm10) genome sequences and annotations were downloaded from the Harvard Medical School Research Computing server (https://rc.hms.harvard.edu). The crystal structure of OC1 DBDs complexed with DNA was obtained from Protein Data Bank under accession code 2D5V. Source data are provided with this paper. MicroRNA expression profiles in different human tissues were obtained from the TissueAtlas web server (https://ccb-web.cs.uni-saarland.de/tissueatlas). MicroRNA target prediction was performed using datasets provided in TargetScan (https://www.targetscan.org).

Research involving human participants, their data, or biological material

Policy information about studies with human participants or human data. See also policy information about sex, gender (identity/presentation), and sexual orientation and race, ethnicity and racism.

Reporting on sex and gender	*Use the terms sex (biological attribute) and gender (shaped by social and cultural circumstances) carefully in order to avoid confusing both terms. Indicate if findings apply to only one sex or gender; describe whether sex and gender were considered in study design; whether sex and/or gender was determined based on self-reporting or assigned and methods used. Provide in the source data disaggregated sex and gender data, where this information has been collected, and if consent has been obtained for sharing of individual-level data; provide overall numbers in this Reporting Summary. Please state if this information has not been collected. Report sex- and gender-based analyses where performed, justify reasons for lack of sex- and gender-based analysis.*
Reporting on race, ethnicity, or other socially relevant groupings	*Please specify the socially constructed or socially relevant categorization variable(s) used in your manuscript and explain why they were used. Please note that such variables should not be used as proxies for other socially constructed/relevant variables (for example, race or ethnicity should not be used as a proxy for socioeconomic status). Provide clear definitions of the relevant terms used, how they were provided (by the participants/respondents, the researchers, or third parties), and the method(s) used to classify people into the different categories (e.g. self-report, census or administrative data, social media data, etc.) Please provide details about how you controlled for confounding variables in your analyses.*
Population characteristics	*Describe the covariate-relevant population characteristics of the human research participants (e.g. age, genotypic information, past and current diagnosis and treatment categories). If you filled out the behavioural & social sciences study design questions and have nothing to add here, write "See above."*
Recruitment	*Describe how participants were recruited. Outline any potential self-selection bias or other biases that may be present and how these are likely to impact results.*
Ethics oversight	*Identify the organization(s) that approved the study protocol.*

Note that full information on the approval of the study protocol must also be provided in the manuscript.

Field-specific reporting

Please select the one below that is the best fit for your research. If you are not sure, read the appropriate sections before making your selection.

[x] Life sciences   [ ] Behavioural & social sciences   [ ] Ecological, evolutionary & environmental sciences

For a reference copy of the document with all sections, see nature.com/documents/nr-reporting-summary-flat.pdf

Life sciences study design

All studies must disclose on these points even when the disclosure is negative.

Sample size	For experiments in cell culture, we have at least 3 biological replicates per condition. For ChIP-seq and ATAC-seq, we have 2 biological replicates per condition. For animal experiments, we infected 60 to 80 animals per group to obtain statistical significance. These samples sizes

	were determined by power analyses using preliminary results showing variation.
Data exclusions	No data were excluded from the analyses
Replication	All data presented are reproducible and representative of at least two independent experiments with multiple replicates except that the high-throughput sequencing experiments were performed once with two or three biologically independent replicates. The high-throughput sequencing experiments were not performed multiple times because they are labor-intensive and time-consuming and their results are consistent with those from other assays such as qPCR and RT-qPCR.
Randomization	Samples and mice were all randomly allocated into experimental groups.
Blinding	The investigators were not blinded to group allocation during data collection or analysis. Blinding was not required because the data are quantitative and do not require subjective analysis.

Reporting for specific materials, systems and methods

We require information from authors about some types of materials, experimental systems and methods used in many studies. Here, indicate whether each material, system or method listed is relevant to your study. If you are not sure if a list item applies to your research, read the appropriate section before selecting a response.

Materials & experimental systems

n/a	Involved in the study	
☐	☒	Antibodies
☐	☒	Eukaryotic cell lines
☒	☐	Palaeontology and archaeology
☐	☒	Animals and other organisms
☒	☐	Clinical data
☒	☐	Dual use research of concern
☒	☐	Plants

Methods

n/a	Involved in the study	
☐	☒	ChIP-seq
☒	☐	Flow cytometry
☒	☐	MRI-based neuroimaging

Antibodies

Antibodies used	Oct-1 antibody (Abcam, ab178869), ONECUT2 antibody (Proteintech, 21916-1-AP), FLAG antibody (Sigma-Aldrich, F1804), ONECUT1 antibody (ABclonal, A12774), β-actin antibody (ABclonal, ACO26), ICP4 antibody (Abcam, ab6514), VP16 antibody (Santa Cruz, sc-7545), histone H3 antibody (Abcam, ab1791), histone H3K9me3antibody (Abcam, ab8898), histone H3K27me3 antibody (Cell Signaling Technology,9733), H3K27ac antibody (Abcam,ab4729), normal rabbit lgG (Merck Millipore, 12-370), FLAG Affinity Gels (Merck Millipore, F2426), HRP-conjugated goat anti-mouse antibody (SouthernBiotech, 1030-05), HRP-conjugated goat anti-rabbit antibody (SouthernBiotech, 4030-05).
Validation	These are all commercially obtained antibodies that had been validated by manufacturers. Validation statements and references can be found in the manufacturers' websites. In particular, the antibodies for ChIP experiments were frequently used in published ChIP experiments. According to the Abcam website (https://www.abcam.com) for histone H3 antibody, ChIP performed in HeLa cells using the H3 antibody (ab1791) showed substantial enrichment of immunoprecipitated DNA at eight different gene loci relative to the negative control, ChIP performed in U2OS cells using the H3K9me3 antibody (ab8898) showed enrichment at six different gene loci relative to the negative control, and ChIP performed in HeLa cells using the H3K27ac antibody (ab4729) showed enrichment at six different gene loci relative to the negative control. According to the Cell Signaling Technology website (https:www.cellsignal.com) ChIP performed in HeLa cells using the H3K27me3 antibody (9733) showed enrichment at four different gene loci. According to the Sigma-Aldrich website (https://www.signaaldrich.com), the supplier validated the anti-FLAG antibody (F1804) by detecting varying amounts of spiked FLAG-tagged protein in CHO lysate. According to the Abcam website, the supplier validated the anti-ICP4 antibody (ab6514) by showing Western blot images using mock-infected cells as negative controls. According to the Santa Cruz website (https://www.scbt.com), the supplier validated the VP16 antibody (sc-7545) by showing Western blot bands from full-length and truncated VP16 proteins. According to the Abclonal website (https://abclonal.com.cn), the supplier shows that Western blotting using the actin antibody (ACO26) shows clear bands at the correct molecular weights from different cells and tissues and that the products have been cited 1723 in references. We also validated OCT-1, ONECUT1 and ONECUT2 antibodies by Western blots following overexpression and/or knockdown experiments. In Supplementary Fig. 5a we show that knockout of OCT-1 results in loss of the OCT-1 specific band and knockdown of ONECUT2 results in reduction of the ONECUT2 specific band at the expected molecular weights. In Supplementary Fig. 5c, we show that transfection of Neuro-2a and 293T cells with ONECUT1 and ONECUT2 expressing plasmids results in appearance of their specific bands at the expected molecular weights.

Eukaryotic cell lines

Policy information about cell lines and Sex and Gender in Research

Cell line source(s)	Vero, 293T, U2OS, HFF, HeLa and Neuro-2a cells were obtained from American Type Culture Collection (ATCC). N2AOct1KO,N2AmiR9 and N2A-C cell lines were derived from Neuro-2a cells in this study as described in Methods.
Authentication	Vero, 293T, U2OS, HFF, HeLa and Neuro-2a cells were obtained from ATCC and frozen in aliquots upon arrival. All cells were

	used under 30 passages from the time of arrival. These cell lines have all been authenticated by ATCC by STR profiling.
Mycoplasma contamination	All cell lines were tested negative for mycoplasma contamination.
Commonly misidentified lines (See ICLAC register)	None

Animals and other research organisms

Policy information about studies involving animals; ARRIVE guidelines recommended for reporting animal research, and Sex and Gender in Research

Laboratory animals	Institute for Cancer Research (ICR) mice from Shanghai Laboratory Animals Center, 6 week old.
Wild animals	No wild animals were used in this study.
Reporting on sex	Sex was not considered in this study. All experiments used male mice.
Field-collected samples	No field-collected samples were used in this study.
Ethics oversight	Mouse housing and experimental procedures were approved by the Laboratory Animal Welfare and Ethics Committee of Zhejiang University in accordance with national guidelines with an approval code of ZJU20220358

Note that full information on the approval of the study protocol must also be provided in the manuscript.

Plants

Seed stocks	*Report on the source of all seed stocks or other plant material used. If applicable, state the seed stock centre and catalogue number. If plant specimens were collected from the field, describe the collection location, date and sampling procedures.*
Novel plant genotypes	*Describe the methods by which all novel plant genotypes were produced. This includes those generated by transgenic approaches, gene editing, chemical/radiation-based mutagenesis and hybridization. For transgenic lines, describe the transformation method, the number of independent lines analyzed and the generation upon which experiments were performed. For gene-edited lines, describe the editor used, the endogenous sequence targeted for editing, the targeting guide RNA sequence (if applicable) and how the editor was applied.*
Authentication	*Describe any authentication procedures for each seed stock used or novel genotype generated. Describe any experiments used to assess the effect of a mutation and, where applicable, how potential secondary effects (e.g. second site T-DNA insertions, mosiacism, off-target gene editing) were examined.*

ChIP-seq

Data deposition

- [x] Confirm that both raw and final processed data have been deposited in a public database such as GEO.
- [x] Confirm that you have deposited or provided access to graph files (e.g. BED files) for the called peaks.

Data access links *May remain private before publication.*	The ChIP-seq data have been deposited to the Gene Expression Omnibus (https://www.ncbi.nlm.nih.gov/geo/) under accession numbers GSE241863.
Files in database submission	Raw data:<br>2-1-Input 1.fq.gz, 2-1-Input 2.fq.gz, 2-1-1P 1.fq.gz, 2-1-1P 2.fq.gz, 2-1-1P 2.fq.gz, 2-2-Input 1.fq.gz, 2-2-Input 2.fq.gz, 2-2-1P 1.fq.gz, 2-2-1P 2.fg.gz<br>Processed data:<br>2-1-Input.sorted.bw, 2-1-IP.sorted.bw, 2-2-Input.sorted.bw, 2-2-IP.sorted.bw
Genome browser session (e.g. UCSC)	None

Methodology

Replicates	We have 2 biological replicates per condition.
Sequencing depth	For the four ChIP-seq samples (two IP samples and two input control samples), the total numbers of reads all were in the range of 36 to 68 million. Alignment efficiencies were around 90% for all samples, so uniquely mapped reads were around the same range. The length of reads was 150 bp and the reads were paired-end.
Antibodies	FLAG Affinity Gels (Merck Millipore, catalog number F2426, lot number SLCF7218)
Peak calling parameters	Reads mapping:<br>$bowtie2_exec --threads 6 --local -q -x ${ref} \ -1 ${sample}_1.clean.fq.gz \ -2 ${sample}_2.clean.fq.gz \ -S ${bam_dir}/

${sample}.sam \   &>${bam_dir}/${sample}_bowtie2_summary.txt
Peak calling for mouse peaks:
macs2 callpeak -g mm \   --qval 5e-2 -B --SPMR \   -f BED -t ../Bam/${sample}.sorted.bed -c ../../${control}/Bam/${control}.sorted.bed \   -n ${sample}_$control --outdir ./peaks/ &>log.macs2
Peak calling virus peaks:
macs2 callpeak -g 150000 \   --nomodel --extsize 147 \   --pval 0.05 \   -f BED -t virus/${sample}.virus.bed -c ../${control}/virus/${control}.virus.bed \   -n ${sample}_${control}.virus --outdir virus/peaks_chip_default/ &>virus/log.macs2

Data quality

About 30,000 peaks are at FDR 5% and above 5-fold enrichment. Also, the results from the two biological replicates are highly consistent.

Software

The peak calling algorithm MACS2 (version 2.2.7.1) was used to identify significant peaks representing DNA binding sites for mouse and virus separately. The peaks were annotated to known genomic features using ChIPseeker (version 1.32.1). Homer2 (version 4.11.1) findMotifsGenome.pl was used to identify enriched known and de novo motifs. Integrative Genomics Viewer (version 2.16.2) was used to visualize read coverages and identified peaks.
